# Core Genome Multilocus Sequence Typing and Single Nucleotide Polymorphism Analysis in the Epidemiology of Brucella melitensis Infections

**DOI:** 10.1128/JCM.00517-18

**Published:** 2018-08-27

**Authors:** Anna Janowicz, Fabrizio De Massis, Massimo Ancora, Cesare Cammà, Claudio Patavino, Antonio Battisti, Karola Prior, Dag Harmsen, Holger Scholz, Katiuscia Zilli, Lorena Sacchini, Elisabetta Di Giannatale, Giuliano Garofolo

**Affiliations:** a National and OIE Reference Laboratory for Brucellosis, Istituto Zooprofilattico Sperimentale dell'Abruzzo e del Molise G. Caporale, Teramo, Italy; b Istituto Zooprofilattico Sperimentale del Lazio e della Toscana M. Aleandri, Rome, Italy; c Department of Periodontology and Restorative Dentistry, Muenster University Hospital, Muenster, Germany; d Bundeswehr Institute of Microbiology, Munich, Germany; University of Iowa College of Medicine

**Keywords:** Brucella melitensis, MLVA, SNP analysis, cgMLST

## Abstract

The use of whole-genome sequencing (WGS) using next-generation sequencing (NGS) technology has become a widely accepted method for microbiology laboratories in the application of molecular typing for outbreak tracing and genomic epidemiology. Several studies demonstrated the usefulness of WGS data analysis through single-nucleotide polymorphism (SNP) calling from a reference sequence analysis for Brucella melitensis, whereas gene-by-gene comparison through core-genome multilocus sequence typing (cgMLST) has not been explored so far. The current study developed an allele-based cgMLST method and compared its performance to that of the genome-wide SNP approach and the traditional multilocus variable-number tandem repeat analysis (MLVA) on a defined sample collection. The data set was comprised of 37 epidemiologically linked animal cases of brucellosis as well as 71 isolates with unknown epidemiological status, composed of human and animal samples collected in Italy. The cgMLST scheme generated in this study contained 2,704 targets of the B. melitensis 16M reference genome. We established the potential criteria necessary for inclusion of an isolate into a brucellosis outbreak cluster to be ≤6 loci in the cgMLST and ≤7 in WGS SNP analysis. Higher phylogenetic distance resolution was achieved with cgMLST and SNP analysis than with MLVA, particularly for strains belonging to the same lineage, thereby allowing diverse and unrelated genotypes to be identified with greater confidence. The application of a cgMLST scheme to the characterization of B. melitensis strains provided insights into the epidemiology of this pathogen, and it is a candidate to be a benchmark tool for outbreak investigations in human and animal brucellosis.

## Abstract (precis)

The use of whole-genome sequencing (WGS) using next-generation sequencing (NGS) technology has become a widely accepted method for microbiology laboratories in the application of molecular typing for outbreak tracing and genomic epidemiology. Several studies demonstrated the usefulness of WGS data analysis through single-nucleotide polymorphism (SNP) calling from a reference sequence analysis for Brucella melitensis, whereas gene-by-gene comparison through core-genome multilocus sequence typing (cgMLST) has not been explored so far.

## INTRODUCTION

Brucellosis is one of the world's most widespread zoonoses, and it is a leading cause of economic losses in production of domestic ruminants (1, 2). Humans can contract the disease by contact with infected animals or their products, with unpasteurized milk being the most common source of brucellosis in urban populations (3, 4). Brucella melitensis, which infects primarily sheep and goats, is the most frequent agent of brucellosis in humans, and it leads to the most severe manifestation of the disease (5).

Due to the high public health and economic burden of brucellosis, European countries have applied surveillance, control, and eradication programs for many years, and most of them have acquired the Officially Brucella melitensis-Free (OBF) status. The disease, however, still persists in several countries in the Mediterranean area. In Italy, despite implementation of the brucellosis eradication program for over 50 years, ovine and caprine brucellosis remains endemic in several southern provinces, in Sicily in particular (6). To date, the regions of Italy still not classified as OBF cover approximately 35.5% of the national land surface, where 39.9% of all small ruminants are farmed (7, 8). The current brucellosis surveillance system in Italy involves regular serological testing and slaughtering of the positive animals from which a bacteriological isolation is performed for confirmation of the diagnosis. Control testing is performed less frequently in the OBF regions, where the goal is to control reintroductions of the disease, whereas it is continuous in the affected areas, where the main aim is eradication of brucellosis.

Efficient and reliable surveillance programs are essential for detection and control of outbreaks and largely depend on collection and access to epidemiological data. Currently, epidemiological investigations rely on the availability of standardized and effective molecular typing methods and analysis tools that allow the public health laboratories to identify and trace an outbreak back to its source.

Identification and typing of B. melitensis are still traditionally performed with the use of biotyping techniques. This methodology, however, suffers from inconsistencies and requires handling of the live bacteria. For this reason, PCR-based typing is now commonly used as an alternative to the culture-dependent typing methods (9–12). The results of the classical biotyping schemes categorize B. melitensis into three biovars that are of limited epidemiological value, as they do not provide sufficient resolution between the isolates. Moreover, an individual biotype often predominates in particular areas, as seen in Italy, where biovar 3 is almost exclusively isolated from the local animal populations (13). B. melitensis is a highly clonal, i.e., monomorphic pathogen, which renders its differentiation at the strain level very difficult (14). Pattern-based techniques such as pulsed field gel electrophoresis and amplified fragment length polymorphism have been applied in the past, but these techniques were not able to differentiate Brucella at the subspecies level, which correlated with low intra- and interlaboratory reproducibility (15). In recent years, the typing methods have shifted toward genome-based approaches that finally allowed an accurate differentiation between Brucella isolates and establishment of a common consensus for the subtyping schemes of this pathogen (6, 16–18).

To date, multilocus variable number of tandem repeats analysis (MLVA) has been considered the most efficient typing method for Brucella spp. Several studies demonstrated that MLVA has a high discriminating resolution, in congruence with MLST, and is sufficient for in-depth study of either genome evolution or outbreak epidemiology (19). According to MLVA schemes, the B. melitensis population can be divided into West Mediterranean, East Mediterranean, and American lineages (20, 21). Moreover, with the development of an international repository, the MLVA data can be stored on web servers and shared between research institutes, thereby increasing MLVA utility as a tool used for analysis of Brucella epidemiology in the world (http://microbesgenotyping.i2bc.paris-saclay.fr/databases/view/907) (22). However, this typing method has several weaknesses, related both to the nature of variable-number tandem repeats (VNTRs) as well as to laboratory demands of the technique itself (12).

With advances in and decreased cost of whole-genome sequencing (WGS), new methods of pathogen typing, including gene-by-gene comparison using core genome multilocus sequence typing (cgMLST), as well as single-nucleotide polymorphism (SNP) calling based on a reference sequence analysis, are considered to be a suitable and more informative replacement of the gold standard typing schemes (23–26). cgMLST is performed by assigning specific alleles to a predefined set of core genes, i.e., genes present in all strains of a given bacterial species. Validated schemes for several pathogens are publicly available and can be shared to ensure reproducibility and comparability of the results across laboratories (23).

The aims of our study were to develop a cgMLST scheme for B. melitensis and to assess the performance of cgMLST and a whole-genome SNP-based approach against the traditional MLVA-16 typing method using a set of animal outbreak-associated isolates and a set of isolates with unknown epidemiological status.

## MATERIALS AND METHODS

### Study design and B. melitensis strains.

**FIG 1:**
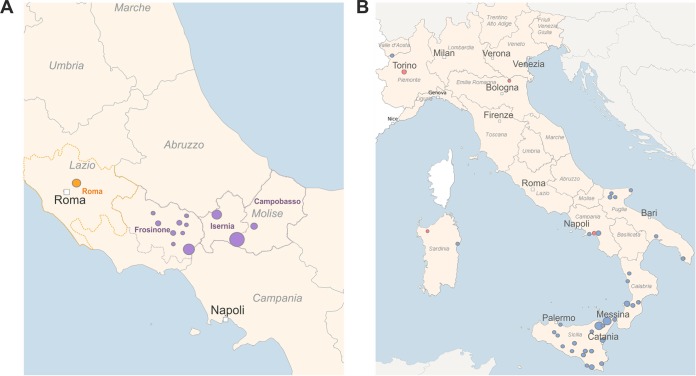
Geographical map for B. melitensis cases studied. (A) epidemiologically related isolates. (B) Isolates with unknown epidemiological status. (A) Separate epidemiological clusters are marked with different colors respective to the provinces of isolation (purple, Frosinone, Isernia, and Campobasso; orange, Rome). (B) The red circles correspond to human isolates and the blue circles to animal isolates.

To evaluate the WGS/NGS approach, we analyzed two different panels of isolates, and we compared the results with those from MLVA-16. The first panel consisted of 37 epidemiologically linked B. melitensis strains isolated during a single outbreak in 21 farms from the provinces of Frosinone, Rome, Isernia, and Campobasso in central Italy (Fig. 1A). The second panel was comprised of 64 isolates of B. melitensis with unknown epidemiological status, collected in Italy from infected livestock between 2011 and 2017 during national eradication program activities, and two related and five unrelated B. melitensis strains isolated from human cases. Figure 1B shows the geographical origin of these samples.

B. melitensis was isolated by following the OIE standard protocol (27). Briefly, animal samples were collected from lymphatic glands (i.e., mandibular, supramammary, and genital lymph nodes), spleen, uterus, or udder, whereas human isolates were obtained directly from blood culture. The isolates were cultured on serum dextrose agar, and the phenotype of the colonies was confirmed using a standard Gram stain and catalase, oxidase, and urease tests. We assigned the Brucella species by PCR, traditional biochemical testing, and serotyping as previously described (13). DNA from the B. melitensis strains was extracted using the Maxwell 16 tissue DNA purification kit (Promega Corporation, Madison, WI) according to the manufacturer's instructions. All isolates were stored at −80°C. Epidemiological data are reported in Table 1.

**TABLE 1:** Brucella melitensis isolates analyzed in this study^*a*^

Sample code	Sample ID	% Good targets for cgMLST	MLVA profile ID	SNP profile ID	Collection date	Farm code	Host species	Region	Province	City	SRA accession no.
Epidemiologically linked isolates											
ItBM_1	2015.IS.2566.1.9	99.4	5	1	14.04.2015	1	Sheep	Molise	Isernia	Rionero Sannitico	SRR6958031
ItBM_2	2015.IS.2547.1.11	99.4	5	2	14.04.2015	1	Sheep	Molise	Isernia	Rionero Sannitico	SRR6958032
ItBM_3	2015.IS.3088.1.42	99.4	1	4	29.04.2015	2	Sheep	Molise	Isernia	Roccamandolfi	SRR6958033
ItBM_4	2015.IS.5088.1.8	99.2	13	5	06.05.2015	3	Sheep	Molise	Campobasso	Bojano	SRR6958034
ItBM_5	2015.TE.21824.1.1	99.3	5	6	16.06.2015	4	Sheep	Lazio	Frosinone	Atina	SRR6958027
ItBM_6	2015.CB.2220.1.19	99.4	3	7	22.03.2015	5	Sheep	Molise	Campobasso	Castropignano	SRR6958028
ItBM_7	2016.TE.17271.1.1	99.4	5	8	06.07.2016	6	Cattle	Lazio	Frosinone	Terelle	SRR6958029
ItBM_8	2015.CB.3742.1.20	99.4	2	9	21.05.2015	5	Sheep	Molise	Campobasso	Castropignano	SRR6958030
ItBM_9	2015.IS.2533.1.11	99.4	5	9	14.04.2015	1	Sheep	Molise	Isernia	Rionero Sannitico	SRR6958035
ItBM_10	2015.IS.3088.1.36	99.4	5	9	29.04.2015	2	Sheep	Molise	Isernia	Roccamandolfi	SRR6958036
ItBM_11	2015.IS.3413.1.7	99.4	5	9	30.04.2015	2	Sheep	Molise	Isernia	Roccamandolfi	SRR6957939
ItBM_12	2015.TE.16173.1.1	99.4	3	9	24.04.2015	7	Goat	Lazio	Frosinone	Sant'apollinare	SRR6957940
ItBM_13	2015.TE.16200.1.1	99.3	3	9	01.06.2015	8	Sheep	Lazio	Frosinone	Frosinone	SRR6957941
ItBM_14	2016.TE.705.1.1	99.4	5	9	22.12.2015	9	Sheep	Lazio	Frosinone	Monte San Giovanni Campano	SRR6957942
ItBM_15	2015.TE.21825.1.1	99.4	5	10	07.07.2015	10	Sheep	Lazio	Frosinone	Casalvieri	SRR6957943
ItBM_16	2015.IS.2529.1.14	99.4	5	11	14.04.2015	1	Goat	Molise	Isernia	Rionero Sannitico	SRR6957944
ItBM_17	2015.TE.16181.1.1	99.4	5	12	24.04.2015	7	Goat	Lazio	Frosinone	Sant'apollinare	SRR6957945
ItBM_18	2014.TE.16510.1.2	99.4	8	13	09.07.2014	11	Goat	Lazio	Frosinone	Roccasecca	SRR6957946
ItBM_19	2015.TE.16142.1.1	99.4	5	14	24.04.2015	12	Sheep	Lazio	Frosinone	Sant'apollinare	SRR6957947
ItBM_20	2015.TE.11849.1.3	99.4	6	15	28.04.2015	13	Sheep	Lazio	Frosinone	Sant'apollinare	SRR6957948
ItBM_21	2015.CB.3742.1.27	99.4	4	16	21.05.2015	5	Sheep	Molise	Campobasso	Castropignano	SRR6957966
ItBM_22	2015.IS.3088.1.30	99.3	7	16	29.04.2015	2	Sheep	Molise	Isernia	Roccamandolfi	SRR6957965
ItBM_23	2015.IS.3681.1.8	99.4	5	16	30.04.2015	2	Sheep	Molise	Isernia	Roccamandolfi	SRR6957968
ItBM_24	2015.TE.16142.1.2	99.4	5	16	24.04.2015	12	Sheep	Lazio	Frosinone	Sant'apollinare	SRR6957967
ItBM_25	2015.TE.16165.1.2	99.4	5	16	24.04.2015	7	Sheep	Lazio	Frosinone	Sant'apollinare	SRR6957962
ItBM_26	2015.TE.16189.1.1	99.4	5	16	05.05.2015	14	Sheep	Lazio	Frosinone	San Donato Val Di Comino	SRR6957961
ItBM_27	2015.TE.16194.1.1	99.4	3	16	05.05.2015	15	Sheep	Lazio	Frosinone	Atina	SRR6957964
ItBM_28	2016.TE.703.1.2	99.4	5	16	22.12.2015	16	Sheep	Lazio	Frosinone	Monte San Giovanni Campano	SRR6957963
ItBM_29	2014.TE.16510.1.7	99.4	9	17	09.07.2014	11	Goat	Lazio	Frosinone	Roccasecca	SRR6957960
ItBM_30	2016.CB.1265.1.7	99.4	5	18	23.02.2016	17	Cattle	Molise	Campobasso	San Massimo	SRR6957959
ItBM_31	2015.IS.6043.1.8	99.4	5	19	24.07.2015	18	Cattle	Molise	Isernia	Cantalupo Nel Sannio	SRR6957977
ItBM_32	2015.IS.5947.1.7	99.4	5	20	22.07.2015	18	Cattle	Molise	Isernia	Cantalupo Nel Sannio	SRR6957978
ItBM_33	2016.TE.17270.1.1	99.3	5	21	16.06.2016	19	Sheep	Lazio	Frosinone	Pontecorvo	SRR6957975
ItBM_34	2015.TE.11843.1.1	99.4	11	22	28.04.2015	20	NA	Lazio	Rome	Rome	SRR6957976
ItBM_35	2015.TE.11845.1.1	99.5	10	22	28.04.2015	21	NA	Lazio	Rome	Rome	SRR6957973
ItBM_36	2015.TE.11847.1.2	99.5	11	22	28.04.2015	20	NA	Lazio	Rome	Rome	SRR6957974
ItBM_37	2015.TE.11847.1.1	99.5	12	23	28.04.2015	20	NA	Lazio	Rome	Rome	SRR6957971
Isolates with unknown epidemiological status											
ItBM_38	2011.TE.19513.1.1	99.4	1	1	2011	NA	Human	Emilia Romagna	Ferrara	Ferrara	SRR6957972
ItBM_39	2011.TE.21031.1.1	99.9	4	2	2011	22	Goat	Sardinia	Nuoro	Orosei	SRR6957969
ItBM_40	2011.TE.3922.1.1	99.6	9	3	2011	23	Goat	Campania	Salerno	Montecorvino Pugliano	SRR6957970
ItBM_41	2011.TE.6299.1.1	99.5	10	4	2011	NA	Human	Campania	Salerno	Salerno	SRR6957984
ItBM_42	2011.TE.1994.1.1	99.6	10	4	2011	23	Sheep	Campania	Salerno	Montecorvino Pugliano	SRR6957983
ItBM_43	2011.TE.6299.1.2	99.6	10	4	2011	NA	Human	Campania	Salerno	Salerno	SRR6957982
ItBM_44	2011.TE.2461.1.1	99.6	10	5	2011	23	Goat	Campania	Salerno	Montecorvino Pugliano	SRR6957981
ItBM_45	2011.TE.12841.1.1	99.3	35	6	2011	24	Sheep	Calabria	Vibo Valentia	Gerocarne	SRR6957988
ItBM_46	2011.TE.12373.1.1	99.4	36	7	2011	25	Sheep	Calabria	Vibo Valentia	Rombiolo	SRR6957987
ItBM_47	2011.TE.12372.1.1	99.4	36	8	2011	26	Sheep	Calabria	Vibo Valentia	Zungri	SRR6957986
ItBM_48	2011.TE.12849.1.1	99.4	36	9	2011	27	Sheep	Calabria	Vibo Valentia	Mileto	SRR6957985
ItBM_49	2011.TE.13541.1.1	99.4	37	10	2011	28	Goat	Sicily	Catania	Caltagirone	SRR6957980
ItBM_50	2016.TE.6344.1.1	99.4	42	11	2016	NA	Human	Sardinia	NA	NA	SRR6957979
ItBM_51	2012.TE.24226.1.1	99.4	42	12	2012	29	Sheep	Sicily	Catania	Mineo	SRR6957993
ItBM_52	2012.TE.24240.1.1	99.4	42	12	2012	30	Sheep	Sicily	Catania	Mineo	SRR6957994
ItBM_53	2013.TE.15028.1.1	98.9	48	13	2013	31	Sheep	Sicily	Caltanissetta	Niscemi	SRR6957995
ItBM_54	2011.TE.4496.1.1	99.3	31	14	2011	32	Sheep	Sicily	Ragusa	Scicli	SRR6957996
ItBM_55	2011.TE.11814.1.1	99.3	24	15	2011	32	Sheep	Sicily	Ragusa	Scicli	SRR6957989
ItBM_56	2011.TE.11815.1.1	99.2	30	15	2011	33	Sheep	Sicily	Messina	San Pier Niceto	SRR6957990
ItBM_57	2011.TE.11821.1.1	99.3	30	16	2011	34	Sheep	Sicily	Messina	Santa Lucia Del Mela	SRR6957991
ItBM_58	2011.TE.4484.1.1	99.4	32	17	2011	35	Sheep	Sicily	Agrigento	Ravanusa	SRR6957992
ItBM_59	2011.TE.744.1.1	99.3	50	18	2011	36	Cattle	Puglia	Foggia	Apricena	SRR6957997
ItBM_60	2011.TE.6840.1.1	99.3	43	19	2011	37	Sheep	Puglia	Taranto	Massafra	SRR6957998
ItBM_61	2011.TE.4500.1.1	99.3	38	20	2011	38	Sheep	Sicily	Messina	Messina	SRR6958008
ItBM_62	2011.TE.11798.1.1	99.3	51	20	2011	39	Sheep	Sicily	Messina	Messina	SRR6958007
ItBM_63	2013.TE.15003.1.1	99.3	44	21	2013	40	Sheep	Sicily	Catania	San Michele Di Ganzaria	SRR6958010
ItBM_64	2011.TE.11842.1.1	99.3	29	22	2011	41	Sheep	Sicily	Messina	Montalbano Elicona	SRR6958009
ItBM_65	2013.TE.15021.1.1	98.9	28	23	2013	42	Sheep	Sicily	Messina	Barcellona Pozzo Di Gotto	SRR6958012
ItBM_66	2011.TE.11802.1.1	99.4	29	24	2011	43	Goat	Sicily	Messina	Barcellona Pozzo Di Gotto	SRR6958011
ItBM_67	2011.TE.11782.1.1	99.4	41	25	2011	44	Goat	Sicily	Catania	Aci Bonaccorsi	SRR6958014
ItBM_68	2013.TE.15029.1.1	99.1	39	26	2013	45	Cattle	Sicily	Messina	Cesaro'	SRR6958013
ItBM_69	2011.TE.21687.1.1	99.4	33	27	2011	46	Sheep	Calabria	Catanzaro	Petrizzi	SRR6958016
ItBM_70	2013.TE.15016.1.1	98.9	26	28	2013	47	Sheep	Sicily	Palermo	Corleone	SRR6958015
ItBM_71	2011.TE.1169.1.1	99.5	46	29	2011	48	Goat	Calabria	Vibo Valentia	Pizzoni	SRR6958039
ItBM_72	2011.TE.1171.1.1	99.5	46	30	2011	49	Sheep	Calabria	Vibo Valentia	Briatico	SRR6958040
ItBM_73	2011.TE.1164.1.1	99.5	47	31	2011	50	Sheep	Calabria	Catanzaro	Chiaravalle Centrale	SRR6958037
ItBM_74	2011.TE.7556.1.1	99.4	34	32	2011	51	Sheep	Puglia	Lecce	Taviano	SRR6958038
ItBM_75	2011.TE.2299.1.1	99.5	45	33	2011	52	Sheep	Puglia	Lecce	Ugento	SRR6958043
ItBM_76	2011.TE.11793.1.1	99.4	40	34	2011	53	Goat	Sicily	Caltanissetta	Caltanissetta	SRR6958044
ItBM_77	2011.TE.11791.1.1	99.4	49	35	2011	54	Sheep	Sicily	Siracusa	Noto	SRR6958041
ItBM_78	2015.TE.26270.1.1	99.4	5	36	2015	NA	Human	Piedmont	Turin	Turin	SRR6958042
ItBM_79	2011.TE.11789.1.1	99.4	6	37	2011	55	Sheep	Sicily	Ragusa	Santa Croce Camerina	SRR6958045
ItBM_80	2013.TE.13528.1.1	98.0	7	38	2013	56	Sheep	Sicily	Messina	Barcellona Pozzo Di Gotto	SRR6958046
ItBM_81	2013.TE.15005.1.1	99.5	27	39	2013	57	Sheep	Sicily	Agrigento	Aragona	SRR6958026
ItBM_82	2012.TE.18485.1.1	99.5	19	40	2012	58	Sheep	Sicily	Caltanissetta	Caltanissetta	SRR6958025
ItBM_83	2011.TE.11828.1.1	99.4	17	41	2011	59	Cattle	Sicily	Messina	Montalbano Elicona	SRR6958024
ItBM_84	2013.TE.15019.1.1	98.2	20	42	2013	60	Sheep	Sicily	Messina	Santa Lucia Del Mela	SRR6958023
ItBM_85	2011.TE.4491.1.1	99.5	23	43	2011	61	Sheep	Sicily	Messina	San Pier Niceto	SRR6958022
ItBM_86	2011.TE.11844.1.1	99.4	22	44	2011	62	Sheep	Sicily	Messina	Montalbano Elicona	SRR6958021
ItBM_87	2011.TE.11805.1.1	99.4	22	45	2011	63	Cattle	Sicily	Messina	Floresta	SRR6958020
ItBM_88	2011.TE.11803.1.1	99.5	21	46	2011	64	Goat	Sicily	Messina	Montalbano Elicona	SRR6958019
ItBM_89	2011.TE.4488.1.1	99.5	24	47	2011	65	Goat	Sicily	Messina	Montalbano Elicona	SRR6958018
ItBM_90	2011.TE.4480.1.1	99.5	24	48	2011	65	Sheep	Sicily	Messina	Montalbano Elicona	SRR6958017
ItBM_91	2011.TE.11810.1.1	99.5	24	49	2011	66	Goat	Sicily	Ragusa	Scicli	SRR6957951
ItBM_92	2011.TE.4467.1.1	99.5	8	50	2011	67	Sheep	Sicily	Siracusa	Noto	SRR6957952
ItBM_93	2011.TE.4471.1.1	99.4	15	50	2011	68	Sheep	Sicily	Palermo	Casteldaccia	SRR6957953
ItBM_94	2011.TE.4474.1.1	99.5	15	50	2011	69	Sheep	Sicily	Catania	Aci Catena	SRR6957954
ItBM_95	2011.TE.4479.1.1	99.5	15	50	2011	70	Sheep	Sicily	Caltanissetta	Niscemi	SRR6957955
ItBM_96	2011.TE.4486.1.1	99.4	15	50	2011	71	Sheep	Sicily	Palermo	Prizzi	SRR6957956
ItBM_97	2011.TE.11826.1.1	99.5	12	51	2011	69	Sheep	Sicily	Catania	Aci Catena	SRR6957957
ItBM_98	2011.TE.4478.1.1	99.5	15	52	2011	72	Sheep	Sicily	Messina	Novara Di Sicilia	SRR6957958
ItBM_99	2017.TE.3072.1.1	99.3	18	53	2017	NA	Human	Piedmont	Turin	Turin	SRR6957949
ItBM_100	2016.TE.6008.1.1	99.5	11	54	2016	NA	Ibex	Aosta Valley	Aosta	Gran Paradiso National Park	SRR6957950
ItBM_101	2011.TE.6837.1.1	99.7	13	55	2011	73	Sheep	Puglia	Foggia	Vieste	SRR6958006
ItBM_102	2011.TE.6838.1.1	99.7	13	55	2011	74	Sheep	Puglia	Foggia	Rignano Garganico	SRR6958005
ItBM_103	2011.TE.6839.1.1	99.7	14	55	2011	75	Sheep	Puglia	Foggia	San Severo	SRR6958004
ItBM_104	2011.TE.6844.1.1	99.7	16	56	2011	75	Sheep	Puglia	Foggia	San Severo	SRR6958003
ItBM_105	2011.TE.1995.1.1	99.6	25	57	2011	76	Goat	Campania	Salerno	Ravello	SRR6958002
ItBM_106	2011.TE.6057.1.1	99.8	3	58	2011	77	Sheep	Calabria	Cosenza	San Lucido	SRR6958001
ItBM_107	2011.TE.6076.1.1	99.7	3	59	2011	78	Cattle	Calabria	Cosenza	Mongrassano	SRR6958000
ItBM_108	2013.TE.2547.1.1	98.7	2	60	2013	NA	Human	Piedmont	Turin	Turin	SRR6957999

a NA, not available.

### MLVA.

Samples were genotyped using the MLVA-16 panel described by Le Flèche et al. (16). Briefly, to assign specific alleles, DNA extracted from each isolate was amplified by multiplex PCR using primers specific for each MLVA-16 locus as described before (12, 16). The amplicons were then separated by capillary electrophoresis using an ABI 3500 instrument with POP 7 polymer, and the allele types were assigned using Genemapper 4.1 (Applied Biosystems, Carlsbad, CA).

### Whole-genome sequencing.

Total genomic DNA was quantified with the Qubit fluorometer (QubitTM DNA HS assay; Life Technologies, Thermo Fisher Scientific, Inc.), and library preparation was performed using the Nextera XT library preparation kit (Illumina Inc., San Diego, CA) or Kapa high-throughput library preparation kit (Kapa Biosystems, Wilmington, MA) according to the manufacturers' instructions. The libraries were sequenced using the Illumina NextSeq 500 platform, producing 150-bp paired-end reads, or Illumina MiSeq, producing 250-bp paired-end reads. After demultiplexing and removal of adapters, reads were trimmed from 5′ and 3′ ends to discard the nucleotides with quality scores of less than 20. Reads shorter than 70 bp and average Phred mean quality of <24 were automatically discarded. Read coverage ranged from 18× to 356×, with an average of 155×. All scaffolds were assembled with SPAdes version 3.11.1 with the –careful option selected (28, 29).

### cgMLST target definition.

To determine the cgMLST gene set, we performed a genome-wide gene-by-gene comparison using the cgMLST Target Definer (version 1.4) function of the SeqSphere+ software, v5.0.90 (Ridom GmbH, Münster, Germany), with default parameters. These parameters comprised the following filters to exclude certain genes of the B. melitensis bv. 1 strain 16M reference genome (NC_003317.1 and NC_003318.1) from the cgMLST scheme: a minimum length filter that discards all genes shorter than 50 bp, a start codon filter that discards all genes that contain no start codon at the beginning of the gene, a stop codon filter that discards all genes that contain no stop codon or more than one stop codon or if the stop codon is not at the end of the gene, a homologous gene filter that discards all genes with fragments that occur in multiple copies within a genome (with identity of 90% and more than 100-bp overlap), and a gene overlap filter that discards the shorter gene from the cgMLST scheme if the two genes affected overlap by >4 bp. The remaining genes were then used in a pairwise comparison using BLAST, version 2.2.12 (parameters used were the following: word size, 11; mismatch penalty, −1; match reward, 1; gap open costs, 5; gap extension costs, 2), with the query chromosomes of one representative for each of the other two B. melitensis biovars (B. melitensis bv. 2 strain 63/9 [NZ_CP007788.1 and NZ_CP007789.1] and B. melitensis bv. 3 strain Ether [NZ_CP007761.1 and NZ_CP007760.1]) (30). Using all genes of the reference genome that were common in all query genomes, with a sequence identity of ≥90% and 100% overlap and with the genome filters start codon filter, stop codon filter, and stop codon percentage filter turned on, the final cgMLST scheme was formed. Therefore, all genes having no start or stop codon in one of the query genomes, as well as genes that had internal stop codons in more than 20% of the query genomes, were discarded.

### SNP analysis.

SNPs were identified using In Silico Genotyper (ISG), version 0.16.10-3 (31). We used default filters to remove SNPs from duplicated regions, minimum quality was set to Phred 30, and the minimum allele frequency was set to 90% in all samples. We used the ISG pipeline with BWA-MEM (version 0.712-r1039) (32) as the aligner and GATK (version 3.9) (33) as the SNP caller. The SNPs were called based on alignment to the reference Brucella melitensis bv. 1 strain 16M (GenBank accession numbers NC_003317.1 and NC_003318.1). Clean unique variants used in further analysis are listed in Tables S1 and S2 in the supplemental material.

### Clustering analyses and cluster definition.

MLVA-16 allelic profiles and SNP matrix data were analyzed using the goeBURST algorithm implemented in PHYLOViZ, version 2.0 (34). Minimum spanning trees (MST) were created using default software settings. The cgMLST profiles were assigned using B. melitensis task template in Ridom SeqSphere+ (35, 36). MSTs were created by pairwise comparison of cgMLST target genes. Missing values were ignored in the calculation of distance between pairs of sample profiles. The links between the MST nodes represented the distance between the genotypes. The cluster cutoff value was defined as the maximum pairwise distance found between epidemiologically linked isolates. The maps in Fig. 1A and B were drawn with SeqSphere+ by using GeoNames (http://www.geonames.org) for geocoding and Natural Earth (http://www.naturalearthdata.com) for drawing vector maps.

Comparison of MLVA, cgMLST, and SNP typing results was performed using Simpson's index of diversity (SDI) and adjusted Wallace (AW) test of congruence using an online tool available at http://www.comparingpartitions.info/?link=Tool (37, 38).

### Accession number(s).

All generated data (Table 1) were submitted to the National Center for Biotechnology Information (NCBI) under the BioProject accession number PRJNA448825 (https://www.ncbi.nlm.nih.gov/bioproject/PRJNA448825).

## RESULTS

### Epidemiologically linked B. melitensis isolates.

The outbreak-related isolates were detected in 21 different farms in three Italian provinces over a period of 1.5 years. The culture-positive samples belonged to 37 animals that were investigated as a part of the within- and among-farm epidemiological investigation (Fig. 1 and Table 1).

**FIG 2:**
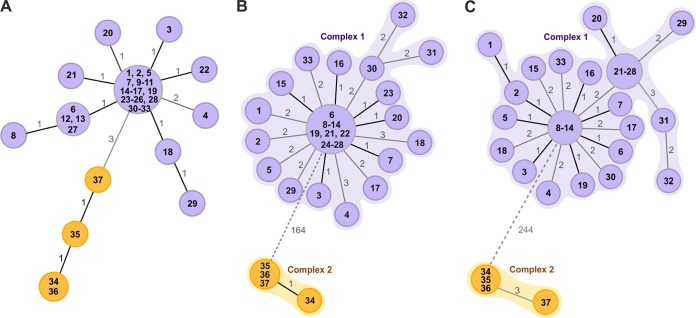
Minimum spanning trees (MST) generated for 37 epidemiologically related isolates. Separate epidemiological clusters are marked with different colors indicating the provinces of isolation (purple, Frosinone, Isernia, and Campobasso; orange, Rome). (A) MST based on B. melitensis MLVA-16 typing. The distance labels correspond to the number of discriminating alleles. (B) MST generated using the gene-by-gene approach. cgMLST profiles were assigned using the B. melitensis task template with 2,704 target genes. The MST was created by cgMLST target pairwise comparison, ignoring missing values, with distance representing the number of diverse alleles. Separate complexes are highlighted. (C) MST based on SNP analysis using B. melitensis strain 16M as a reference. The distance labels correspond to the number of discriminating SNPs between neighboring genotypes. The prefix ItBM was omitted from the isolates' labels for simplicity.

MLVA-16 revealed the presence of 13 different genotypes, divided into two groups formed by single-locus variants and one double-locus variant (Fig. 2A). MST showed that the groups were split by mutations in the three hypervariable loci bruce04, bruce09, and bruce16. One group included three genotypes of four isolates collected from farms located in the province of Rome, whereas in the remaining 33 strains from Isernia, Campobasso, and Frosinone provinces we identified 10 distinct genotypes.

We generated a cgMLST scheme comprised of 2,704 targets based on the B. melitensis 16M reference genome. The cgMLST clustering divided the isolates into two different genetic complexes, grouping the two farms from the province of Rome (complex 2) separately from the remaining 19 farms (complex 1). The genetic division measured with the cgMLST panel was for 164 different genes (Fig. 2B). The analysis using the B. melitensis panel found one prevalent genotype that was similar across the provinces of Frosinone, Campobasso, and Isernia and was found in 10 of the tested farms.

Sixteen isolates in complex 1 shared identical core genome profiles, and the largest distance between any two neighboring isolates was not greater than three genes. In complex 2, one isolate was separated from the other three by one gene difference.

Removing 50 targets from the analysis where any value was missing decreased the distances between the nodes even further and classified all samples from Rome as identical (not shown). A within-farm genetic variation was also observed.

The SNP analysis identified 3,390 SNPs, of which 3,146 were classified as clean unique variants and included in further analysis. The tree split the samples into two genetic clusters with a distance of 244 SNPs between them (Fig. 2C). We observed a within-farm variation of 2 MLVA-16 loci, 3 cgMLST loci, and 4 SNPs. The maximum pairwise distance found in the two complexes was 6 cgMLST genes and 7 SNPs.

The comparison of discriminatory power of MLVA, cgMLST, and SNP typing showed that the SNP-based approach was superior to the other two methods, with an SDI of 0.922 and 95% confidence intervals (CI) of 0.866 to 0.978. SDI of cgMLST was calculated to be 0.815 (95% CI, 0.685 to 0.945), and SDI of MVLA-16 was 0.674 (95% CI, 0.505 to 0.843). SNP typing was a good predictor of cgMLST, with an AW of 0.788 (95% CI, 0.546 to 1.000). The correspondence of the typing results, however, was not bidirectional, as the cgMLST to SNP AW was 0.295 (95% CI, 0.136 to 0.453). Comparison of the remaining pairs of typing schemes showed that there was no congruence between clusters they predicted (the AW of each pair did not exceed 0.03).

### B. melitensis isolates with unknown epidemiological status.

**FIG 3:**
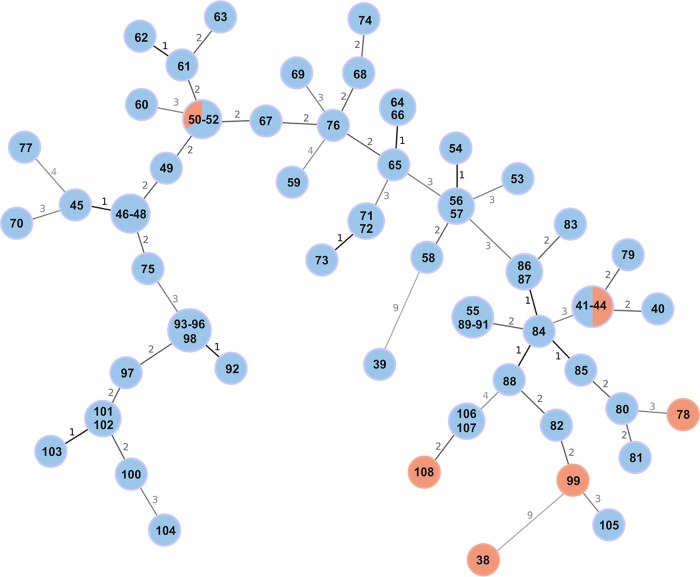
Minimum spanning tree (MST) based on B. melitensis MLVA-16 typing results generated for 71 isolates with unknown epidemiological status. The tree was generated using the goeBURST algorithm in PHYLOViZ software. The distance labels correspond to the number of discriminating alleles. The red nodes correspond to human isolates and the blue nodes to animal isolates. The prefix ItBM was omitted from the isolates' labels for simplicity.

MST calculated using the MLVA-16 typing results showed a distance between directly linked nodes not exceeding 9 VNTR loci (Fig. 3). Fifty-one MLVA-16 profiles were assigned to the 71 strains, and diverse allele variants were identified in all loci apart from bruce45. Eleven profiles were shared by more than one isolate, which, with the exception of one human isolate, corresponded to the samples originating from the same geographical location (Table 1).

MLVA profiles tend to be conserved between epidemiologically linked strains; therefore, the strains from an outbreak are likely to have a similar MLVA profile. Three MLVA-16 profiles, 10 (samples ItBM_41 to ItBM_44), 15 (samples ItBM_93 to ItBM_96 and ItBM_98), and 24 (ItBM_55 and ItBM_89 to ItBM_91), were identified in more than three strains, suggesting close relatedness of samples within these profiles. The method also allowed identification of two clear outliers. Samples ItBM_38 and ItBM_39 showed a distance of 9 alleles from the nearest B. melitensis isolate and no relatedness to one another.

According to our MLVA-16 data, only three out of six human cases could be linked to a specific animal source analyzed in our study. Human samples ItBM_41 and ItBM_43, isolated from two patients in the city of Salerno, shared the same MLVA-16 profile as two animal isolates from a farm in Salerno province (samples ItBM_42 and ItBM_44), all collected in 2011. Human isolate ItBM_50 and two animal isolates (ItBM_51 and ItBM_52) were assigned MLVA-16 profile 42, but interestingly, ItBM_50 was isolated 4 years later than the animal strains. The other three human samples did not show sufficient relatedness to any of the animal isolates to reliably trace the source of infection. The number of variable loci, in these cases, ranged from 2 to 9 in relation to the closest neighboring MLVA-16 profile.

**FIG 4:**
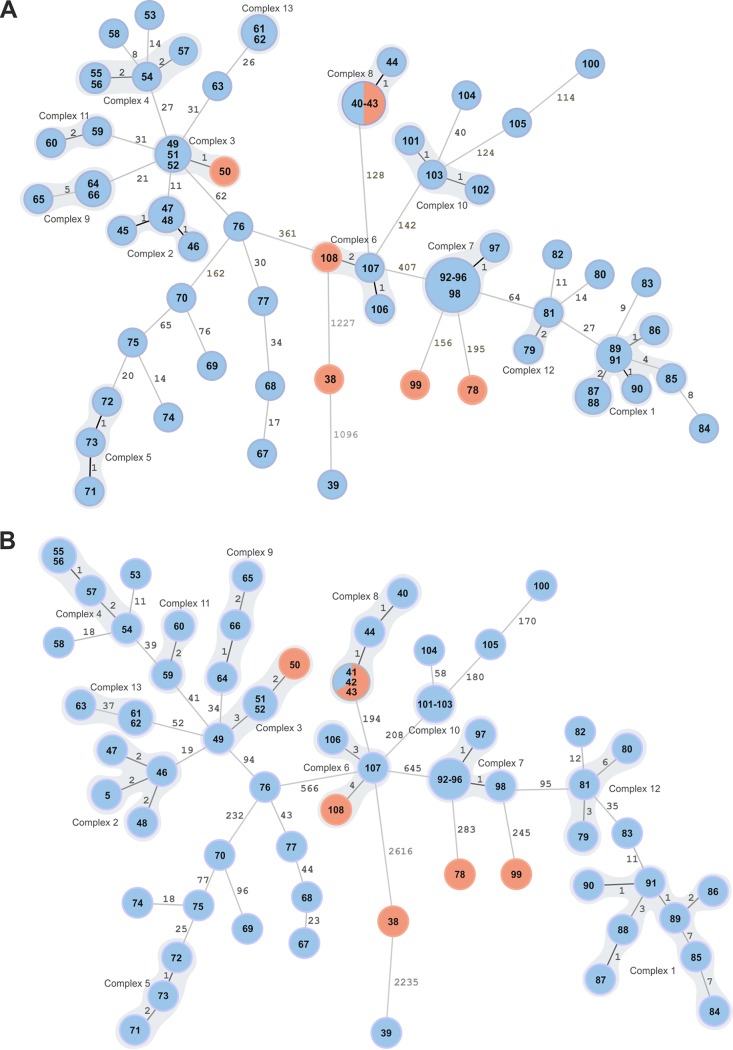
Minimum spanning trees (MST) based on WGS analysis results generated for 71 isolates with unknown epidemiological status. (A) MST generated using gene-by-gene approach. cgMLST profiles were assigned using B. melitensis task template with 2,704 target genes. The MST was created by cgMLST target pairwise comparison, ignoring missing values, with distance representing the number of diverse alleles. Separate complexes are highlighted. (B) MST based on SNP analysis using B. melitensis strain 16M as a reference. The distance labels correspond to the number of discriminating SNPs between neighboring genotypes. The red color nodes correspond to human isolates and the blue nodes to animal isolates. The prefix ItBM was omitted from the isolates' labels for simplicity.

To increase the discriminatory power of the investigation, we analyzed 71 assemblies using a cgMLST scheme. The genome assemblies exceeded 98% of good targets (with a mean of 99.4%). Isolates ItBM_38 and ItBM_39 were clear outliers, separated from the closest neighbor by 1,227 genes, and 1,096 loci from one another (Fig. 4A).

Based on the analysis of epidemiologically related isolates, we used 6-gene difference as a threshold for a potential complex of related cases. Thirteen complexes were assigned in the MST data analysis. Gene-by-gene analysis confirmed relatedness of genotypes with MLVA-16 profiles 10 and 15; however, according to cgMLST two other isolates were at a distance of 0 to 1 gene away from the samples of MLVA-16 profile 15, as was one other isolate of profile 10. ItBM_55, classified as MLVA-16 profile 24, was shown not to be closely linked to other isolates with the same MLVA-16 alleles when examined with a gene-by-gene approach.

Using cgMLST, four of the human isolates (ItBM_41, ItBM_43, ItBM_50, and ItBM_108) were found in the distance not exceeding 2 alleles to the closest animal strain. Two of the human samples originating in Piedmont (ItBM_99 and ItBM_78) were genetically different from the animal samples, with 156 and 195 allele differences from the closest isolate, and could be identified as outliers, although they were distantly related to other Italian genotypes. Divergence of these two samples was not evident in MLVA-16 typing (distance of 2 to 3 alleles to other isolates).

A total of 6,540 SNPs were discovered by mapping 71 genomes to the B. melitensis 16M reference strain. Out of these, 6,027 were considered high-quality discriminatory SNPs and were used to infer the relationship between the strains. We applied the threshold of 7 SNPs to detect the clusters of closely related cases, and in accordance with cgMLST analysis, we identified 13 complexes (Fig. 4B). The highest distances observed between two adjoining isolates were 2,616 and 2,235, belonging to the SNP profiles of ItBM_38 and ItBM_39, which also were marked as outliers by MLVA-16 and cgMLST analyses.

In agreement with cgMLST, two human cases (ItBM_78 and ItBM_99) could not be traced to any of the analyzed animal strains of B. melitensis, and both differed by more than 200 SNPs from the nearest SNP profile. Close genetic relationship to at least one isolate from an animal host was confirmed for ItBM_41, ItBM_43, ItBM_108, and ItBM_50.

The SDI for the three typing schemes were calculated to be 0.986 (95% CI, 0.978 to 0.995) for MLVA-16, 0.988 (95% CI, 0.978 to 0.998) for cgMLST, and 0.992 (95% CI, of 0.985 to 1.000) for SNP typing. AW test showed the highest congruence between SNP- and cgMLST-based clusters when the SNP method was used as a primary typing method (AW of 0.840; 95% CI, 0.753 to 0.927). When we used cgMLST as the primary method, however, the AW value dropped to 0.573 (95% CI, 0.290 to 0.856). MLVA-16 was a poor predictor of SNP (AW of 0.318; 95% CI, 0.112 to 0.524) and of cgMLST (AW of 0.494; 95% CI, 0.333 to 0.655).

## DISCUSSION

Our study compared the performance of two WGS-based typing methods, SNP analysis and cgMLST, with the gold standard MLVA-16 in an analysis of the phylogenetic relationship between isolates of B. melitensis detected in the context of a national surveillance program.

We found that all three typing schemes generally performed equally, and although SNP analysis had the highest resolving power in terms of differences detected between the isolates, the number of predicted genotypes in the surveillance scenario was comparable for all examined methods (51 MLVA-16 types, 55 cgMLST types, and 60 SNP types), and the SDI were similar. However, SDI test applied to samples from epidemiologically linked sets showed that SNP analysis was superior in differentiating between closely related samples. This suggests that while WGS-based approaches could be used as standalone tools in establishing phylogenetic relationships, MLVA-16 optimally should be supported by either SNP or gene-by-gene typing results.

In our study, all three typing methods accurately predicted the presence of two genomes divergent from the rest of the Italian strains. Indeed, the majority of analyzed samples belonged to the West Mediterranean lineage of B. melitensis, while the outliers were members of the East Mediterranean and American lineages (6). Epidemiological investigation showed that ItBM_38 was isolated from a Syrian patient with a history of frequent travel to his home country, where the same East Mediterranean lineage is thought to be prevalent (39). The strain ItBM_39, on the other hand, was isolated from a goat imported to Italy from Spain.

Two human isolates, ItBM_50 and ItBM_108, were found in the same SNP and cgMLST complexes as animal strains, but interestingly, the samples were collected a few years apart and in different geographical locations, suggesting that animal isolates could have been closely related (or ancestral) to the source of human infection but not directly involved in the transmission event. In these cases, observation based on WGS typing indicates that strains of B. melitensis were circulating in the affected regions of Italy for many years and the surveillance program failed to eradicate them.

For distantly related genomes from the same lineage, cgMLST as well as SNP analysis provided higher phylogenetic distance resolution than MLVA-16, and therefore spotting divergent genotypes unlikely to be connected to the other circulating strains was possible with greater confidence. This was particularly apparent in the case of two clinical isolates (ItBM_99 and ItBM_78) and in the case of B. melitensis collected from an ibex (Capra ibex ibex) in Gran Paradiso National Park, located in the Graian Alps in Italy (sample ItBM_100). This demonstrated that while all applied schemes could be used to identify very distant genomic outliers within the Brucella population, WGS-based schemes were superior in identifying unrelated cases belonging to the same lineage. Additionally, within the clusters of similar genotypes, cgMLST performed equally to the SNP analysis, but some discrepancies were observed in MLVA-16 analysis. For instance, seven isolates from Sicily had profiles differing by a maximum of two SNPs or one gene (samples ItBM_92-ItBM_98), suggesting that they were very closely related. However, while five of these isolates shared MLVA-16 profile 15, one belonged to type 8 (1 allele distant; bruce19) and another to type 12 (2 alleles distant; bruce4 and bruce7). The interpretation of WGS results therefore suggests that these were actually strains from the same complex, while MLVA-16 typing would not necessarily lead to the same conclusion. A similar observation was reported by Dallman et al. (40), who showed that using SNP analysis of E. coli O157 isolates identified linked cases with twice the sensitivity of the MLVA-16 scheme, while Georgi and colleagues (39) demonstrated that MLVA-16 had lower discriminatory power than the WGS-based SNP typing by analyzing a set of 63 human B. melitensis isolates. Interestingly, in our cluster of outbreak-related cases, we identified several genotypes that differed by one, two, or three hypervariable alleles and belonged to an outbreak caused by a single epidemic clone. WGS-based analysis of these strains showed that they were very closely related (up to 6 genes or 7 SNPs of difference). Together, these observations show that MLVA-16 profiling might not provide enough resolution to accurately predict phylogenetic relationships between isolates involved in an ongoing outbreak or strains that have been circulating over the years with no direct link to one another.

SNP analysis has successfully been used to discriminate between Brucella species and to map the geographic distribution and global spread of B. melitensis (18, 39, 41). However, to date there is no official and validated cgMLST scheme for any of the Brucella species. Consequently, the cluster types for specific data and particularly for closely related strains can only be assessed empirically and therefore are subject to variation between laboratories. In order to reliably interpret the results, cutoff values first should be established based on the analysis of a significant number of closely related strains and unrelated strains sharing common or closely related profiles assigned using gold standard typing methods. The analysis of outbreak-related isolates suggested that two independent epidemic clones were circulating in central Italy at the same time. The maximum pairwise distance between isolates within complexes formed by these clones did not exceed 6 genes (cgMLST) or 7 SNPs. These findings highlight the potential criteria necessary for inclusion of an isolate into a brucellosis outbreak cluster that we would therefore suggest to be ≤6 loci in the cgMLST and ≤7 in WGS SNPs analysis.

Jackson et al. argued that a general cutoff value applied in SNPs or cgMLST could not always reliably predict whether samples were epidemiologically related and that isolates with SNP differences ranging from 10 to 30 were frequently linked (42). Thus, we believe that the proposed cutoff values should be taken as a guideline and interpreted in the context of available epidemiological information.

Using a typing approach that offers maximum resolution is particularly important for tracing the spread of a disease during an outbreak. SNP analysis potentially has the highest discriminatory power among the typing methods, as polymorphisms can be discovered in both coding and noncoding regions of the genome. However, the choice of a reference genome can significantly influence the number of identified SNPs and the accuracy of the reconstructed phylogenetic relationships (43). cgMLST relies on the availability of complete, accurately sequenced genomes for the generation of the typing schemes. Inclusion of coding sequences only decreases the number of sites typed in the analysis, but at the same time it facilitates standardization and reproducibility of the analyses as it focuses on a predefined set of genes. In WGS analysis the quality of the reads as well as of the assembly plays a crucial role in achieving reliable cgMLST results. While in our study all samples reached at least 98% of good targets, low-quality assemblies are likely to have a reduced number of good targets and therefore lead to generation of inaccurate results in phylogenetic analysis. We therefore propose that the data with good targets of less than 97% should be taken with caution.

In conclusion, WGS/NGS data can be used effectively to gain a better understanding of epidemiology and dynamics of Brucella populations and to gather in-depth information which can be used for source tracing in case of outbreaks within animal holdings, zoonotic or foodborne infections, and illegal animal movements. Moreover, WGS data facilitate the assessment of the possible extent of an ongoing outbreak and the reliable prediction of the routes of its spread.

In accordance with the One Health approach, public health agencies can implement WGS to aid in disease control and eradication plans. In our study, both cgMLST and SNP analysis performed well despite the restricted level of B. melitensis genetic diversity, and we demonstrated that the performance of the gene-by-gene approach was comparable to that of the SNP analysis. On the basis of these results, we believe that MLVA-16 typing of B. melitensis in Italy can now be successfully replaced by the more informative WGS analysis.

## Supplementary Material

Supplemental file 1

Supplemental file 2
